# Traditional rye varieties exhibit drought tolerance traits but maintain lower yields than modern varieties under drought stress

**DOI:** 10.1038/s41598-026-51544-5

**Published:** 2026-05-05

**Authors:** Marcela Hlaváčová, Karel Klem, Jaromír Pytela, Otmar Urban, Natálie Pernicová, Jan Balek, Daniela Semerádová, Milan Fischer, Reimund P. Rötter, Mercy Appiah, Petr Hlavinka, Vladimíra Horáková, Petr Škarpa, Miroslav Trnka

**Affiliations:** 1 Department of Climate Change Impacts on Agroecosystems, Global Change Research Institute of the Czech Academy of Sciences, Bělidla 986/4a, 60300 Brno, Czech Republic; 2 Department of Agrosystems and Bioclimatology, Faculty of AgriSciences, Mendel University in Brno, Zemědělská 1665/1, 61300 Brno, Czech Republic; 3 Laboratory of Ecological Plant Physiology, Global Change Research Institute of the Czech Academy of Sciences, Bělidla 986/4a, 60300 Brno, Czech Republic; 4 Plant Phenotyping and Biotechnology Platform, Photon Systems Instruments, Průmyslová 470, 66424 Drásov, Czech Republic; 5 Department of Matters and Energy Fluxes, Global Change Research Institute of the Czech Academy of Sciences, Bělidla 986/4a, 60300 Brno, Czech Republic; 6 Department of Crop Sciences, Faculty of Agricultural Sciences, Tropical Plant Production and Agricultural Systems Modelling, Georg-August- Universität Göttingen, Wilhelmsplatz 1, 37073 Göttingen, Germany; 7 Plant Production Section, Department of Utility Value Testing, Central Institute for Supervising and Testing in Agriculture, National Plant Variety Office, Hroznová 63/2, 60300 Brno, Czech Republic; 8 Department of Agrochemistry, Soil Science, Microbiology and Plant Nutrition, Faculty of AgriSciences, Mendel University in Brno, Zemědělská 1665/1, 61300 Brno, Czech Republic

**Keywords:** Drought stress, Phenotyping platform, *Secale cereale* L., Yield components, Ecology, Ecology, Plant sciences

## Abstract

Compared with other cereals, rye (*Secale cereale* L.) has traits that contribute to superior drought tolerance, thus making it a suitable alternative under intensifying droughts related to climate change. The distributions of these traits across a range of varieties and their relationships with yield potential have yet to be fully elucidated. Therefore, the sensitivity of 20 varieties, which differ in origin and age, to drought stress between the second half of stem elongation and the beginning of grain filling was analyzed. Potted plants were exposed to drought on an automated phenotyping platform, enabling accurate simulation of water loss for all varieties. Soil moisture was reduced to 30% of the soil water capacity, followed by rewetting. Although traditional varieties exhibited greater stability of key yield components under drought stress, modern varieties achieved higher absolute yields under both drought and well-watered conditions. Therefore, traditional varieties cannot replace modern varieties but remain valuable donors of drought resistance traits.

**Supplementary Information:**

The online version contains supplementary material available at 10.1038/s41598-026-51544-5.

## Introduction

Drought is the primary abiotic stress factor that limits cereal production worldwide^1,2^, and ongoing climate change is amplifying this constraint by increasing the frequency and intensity of compound drought–heat events across much of Europe^3^. In temperate agroecosystems, key abiotic changes include higher evaporative demand, altered rainfall distribution, progressive soil moisture deficits, and a greater likelihood that stress factors coincide with sensitive phenological stages such as stem elongation, anthesis, and early grain filling^3,4^. These conditions affect the physiological performance of cereals through stomatal closure and reduced carbon assimilation, accelerated canopy senescence, and constraints on post-anthesis nitrogen (N) uptake and N allocation to grains, ultimately reducing yield components (e.g., grain number and thousand-grain weight) and harvest index^5–7^.

Based on both 2024 data and the 2020–2024 mean, rye ranks ninth in terms of global production and fifth within the EU-27 (FAOSTAT Database, http://www.fao.org/faostat/). Rye is primarily cultivated in Eastern, Central, and Northern Europe (especially in Germany and Poland), as well as Canada, Turkey, Australia, Argentina, and other countries stretching within main rye cultivation areas (i.e., northern latitudes in the range of 30–65° and southern latitudes in the range of 30–50°, as presented by Leff et al.^8^. A high proportion of total produced rye grains – approximately 50–75% – are used for bread baking, mostly in European countries that are traditional producers of rye^9^. The rye produced in these regions complements the wheat diet^10^.

Rye (*Secale cereale* L.) can tolerate a wide range of adverse environmental conditions, including poor soils with low nutrient availability, cooler climates, and dry sites Colombo 2022^14,4^. The drought resilience of rye is largely attributed to its deep and extensive root system, as evidenced by traits such as increased root length density, lateral root branching, and enhanced biomass allocation under stress^11^. Among small-grain cereals, rye has the highest overwintering rate and the strongest resilience to drought stress, making it suitable for cultivation even under conditions that are not favorable for the cultivation of any other cereal crop^9^. Moreover, modern hybrid rye varieties have relatively high yield potentials, making them competitive with wheat even in fertile soils^9^. Rye also has the fastest spring growth among winter cereals, even under water-limited conditions, thereby preventing soil erosion^ 9,4^. Due to these features, rye holds promise for sustainable grain production in the face of climate change^10^. Recent studies underline the importance of integrating improved nutrient‑use strategies and stress‑response mechanisms such as enhanced metabolic activity and antioxidant defense into sustainable cereal production systems, which aligns with the role of rye as a climate‑resilient crop^12,13^.

While current cereal breeding methods primarily focus on high and stable yields^9^, traditional landraces are selected for region-specific environmental stress tolerance^14^. This selection process contributes to yield stability^15^ and enhanced resilience to both biotic and abiotic stresses^16–18^. In contrast, modern hybrid varieties often lack competitiveness under such adverse conditions^19^. Therefore, developing hybrid rye varieties with stable yields across diverse environmental conditions^20^ and enhancing the competitiveness of rye under current agricultural systems^21^ are key breeding objectives in the context of climate change. Despite the recognized drought tolerance of rye, little is known about how traditional and modern ryes differ in terms of their tolerance mechanisms, the specific traits underlying this resilience, and how these factors are related to yield performance under both water-sufficient and drought conditions.

Therefore, it is essential to evaluate rye varieties for drought tolerance, as rye is a promising cereal crop that can contribute to sustainable cereal production in the context of climate change. Given that drought stress is the primary abiotic factor causing cereal yield losses worldwide, it is crucial to understand the responses of different rye varieties. Therefore, twenty winter rye varieties from various rye-growing regions were examined to determine their tolerance to drought stress during critical growth stages from the second half of stem elongation to the beginning of grain filling. The main objectives of this study were as follows: (i) to assess differences in yield formation traits and physiological proxies (flag-leaf chlorophyll index and grain δ^13^C and δ^15^N) between traditional and modern winter rye varieties in response to drought, and (ii) to determine whether traditional rye varieties are more productive than modern varieties under drought conditions. This study aimed to test the following hypotheses: (1) traditional varieties are more productive under drought stress conditions, and (2) modern rye varieties are more productive when there is a sufficient water supply.

## Materials and methods

### Plant, soil and growth conditions

Twenty winter rye varieties (*Secale cereale* L.), including both traditional and modern varieties, were selected from countries spanning a wide range of latitudes within the rye belt (see Supplementary Table S1 ). Varieties were classified as traditional (1953–1990) or modern (1991–2018) based on the year of seed registration in the GRIN Czech genebank database; when this information was unavailable, the year of registration in the National Varietal List of the country of origin was used (see Supplementary Table S1). Although the first hybrid variety was bred in Germany as early as 1984^22^, major progress in rye genetics since 1990, driven by the development of DNA marker techniques, has enabled intensive breeding of hybrid varieties and their successful introduction into rye production^23^. Therefore, rye varieties registered between 1953 and 1990 were categorized as traditional (old), whereas those registered between 1991 and 2018 were considered modern in this study. Seeds were provided free of charge by the plant genebank GRIN Czech (https://grinczech.vurv.cz/gringlobal/), except for recently registered varieties KWS Binntto, KWS Vinetto, SU Cossani, SU Performer, and Inspector. These varieties were provided free of charge by the Central Institute for Supervising and Testing in Agriculture (Brno, Czech Republic).

All varieties were sown on 22 October 2018 into 3-L truncated cone plastic pots (height: 20.5 cm, lower base width: 11.5 cm, upper base width: 15.7 cm). Each pot was filled with approximately 2.77 kg (dry mass) of topsoil (≈ 3 L), followed by irrigation to the target SWC levels. The topsoil (0–30 cm) originated from the Polkovice experimental site (199 m a.s.l.; Czech Republic). The soil is classified as Luvic Chernozem with loess as a parent material and a silty clay texture (70% loam, 21% clay, 9% sand). The soil properties were as follows: pH (CaCl_2_) 7.16; the N_tot_ 0.23%; C_tot_ 2.53%; and Mehlich III-extractable Ca, K, Mg, and P contents of 5701, 427, 247, and 84 mg kg^− 1^, respectively.

Two seeds were sown in each pot, but only one plant was retained at the beginning of the vegetative season to ensure sufficient space for optimal growth. The pots were placed on a concrete floor in a vegetation hall at Mendel University in Brno (235 m a.s.l.; 49°12′36.62892′′ N, 16°36′48.64716′′ E). The hall had wire netting instead of walls and a roof, allowing exposure to ambient weather conditions. The experimental vegetation period for the medium-maturing group lasted 260 days (from 22 October 2018 to 8 July 2019). Other varieties showed phenological shifts of − 4 to + 2 ± 1 days relative to the medium-maturing group (see Supplementary Table S1 for details). Throughout the vegetation period, the plants were regularly irrigated to prevent drought stress (87 mm in total per vegetation season) and were fertilized and treated for insect pests and fungal diseases using recommended products (see Supplementary Table S2).

### Experimental phase

At the beginning of the experimental phase, the plants were transported from the vegetation hall to the PlantScreen™ Modular System phenotyping platform (Photon Systems Instruments, Ltd., Drásov, Czech Republic; 265 m a.s.l.; 49°20′14.9′′ N, 16°28′34.1′′ E). The experimental phase commenced at the second half of stem elongation (DC 37–41 according to Zadoks decimal codes^24^). Prior to the experimental phase, the plants were acclimated for two days in the greenhouse to adapt to the microclimate. Subsequently, all the plants were irrigated to the same level of 70% soil water capacity (SWC) (day 0 – preexperimental stage, 7 May 2019), corresponding to a soil weight of 3.57 kg.

The plants were then divided into two treatment groups (*n* ≥ 4 per treatment per variety): (1) control plants were irrigated to maintain 70% SWC throughout the experimental phase, while (2) drought-stressed plants were allowed to dry down naturally; after daily weighing, only a small amount of water was added so that each pot reached the SWC of the second-wettest pot within the drought treatment, which served as the daily reference. Soil moisture levels were monitored daily on the phenotypic platform using automated weighing and irrigation. Each pot was weighed in the first round of measurements, irrigated in the second round according to its weight, and weighed again after watering. Thus, irrigation was applied to achieve a defined SWC value: the 70% SWC for the control plants and the SWC of the second-wettest pot within the drought treatment for the drought-stressed plants. When the mean SWC of all drought-stressed pots reached 30% after watering (corresponding to the soil weight of 3.11 kg and to twice the permanent wilting point of the soil used, i.e. 15% SWC, on day 29 of the experimental phase), irrigation of these plants was resumed to match the control treatment (70% SWC) and allow recovery, starting on day 31 of the experimental phase (7 June 2019). The natural drying phase of the drought-stressed plants lasted 28 days from the initial irrigation on day 0 (8 May 2019 to 6 June 2019). Owing to the limited capacity of the phenotyping platform, the number of control plants for two randomly selected rye varieties—one traditional variety (Musketeer) and one modern (Wrens-5)—was reduced by one, resulting in four replications.

**Fig. 1:**
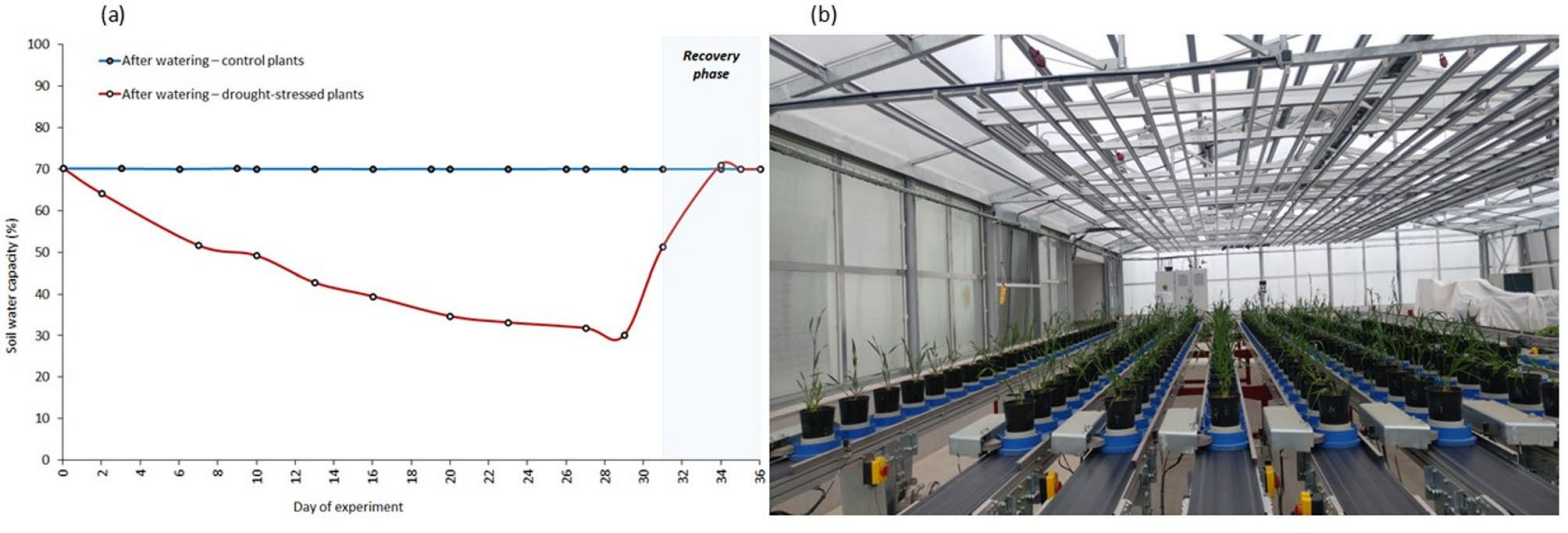
(**a**) Soil water capacity (SWC; %) of the pots during the experiment from 7 May 2019 (day 0) to 12 June 2019 (day 36). On day 0, both drought-stressed and control plants were irrigated to the same level of 70% SWC. The SWC of the drought-stressed plants was subsequently maintained at the level of the second wettest pot within this group. When the driest pot among the drought-stressed plants reached 30% SWC (double the value of the wilting point of 15%) on day 29, reirrigation of the drought-stressed plants started on day 31, restoring the SWC to 70% by day 36. On this day, the mean SWC of the drought-stressed plants matched that of the appropriate control plants. On day 37, all the plants were transported to the vegetation hall. (**b**) Illustrative photo of the phenotyping platform (PlantScreen™ Modular System) used for the experimental phase.

On the 30th day of the experimental phase, the in vivo contents of total chlorophyll (Chl *a* + *b*) in flag leaves were measured using a factory-calibrated Dualex 4 Flav device (Force A, Orsay, France) to assess the leaf senescence rates of both drought-stressed and control plants. The total chlorophyll content (chlorophyll index) was measured in the middle part of a flag leaf – i.e., the first leaf from the top of a plant – in all plants of each variety to ensure consistency of these measurements. The experimental phase was terminated when the mean SWC of the drought-stressed plants equaled that of the control plants (on the 36th day of the experimental phase, corresponding to the beginning of grain filling, DC 71–73; Fig. 1). The recovery stage lasted 6 days (from 31st day to 36th day – from 7 June 2019 to 12 June 2019). The plants were then returned to the vegetation hall on the 37th day and cultivated until manual harvest at full ripening (DC 92). Phenological development was monitored visually during cultivation. Air temperature was recorded at 2 m above ground level in the vegetation hall and above the plant canopy in the PSI Drásov greenhouse. Global radiation and photosynthetically active radiation were measured in the vegetation hall and in the greenhouse of Drásov, respectively. Precipitation during plant cultivation in the vegetation hall was recorded in the nearby arboretum of Mendel University in Brno. Relative air humidity was also measured in Drásov (see Supplementary Fig. S1, S2).

### Assessment of yield traits

The harvested aboveground biomass was dried for 12 h at 105 °C in an automatic drying oven. The aboveground biomass per plant (AB; g) and the combined weight of straw and leaves without spikes (SLW) were subsequently recorded. The spikes were separated, and the grains were manually rolled out to determine yield formation parameters, including the grain number per spike (GN; pcs), grain weight per spike (GW; g), thousand-grain weight (TGW; g; calculated from measured GW and GN), and harvest index (HI; unitless).

### Isotopic analyses

After the assessment of yield formation parameters, grain samples were analyzed at the Laboratory of Ecological Plant Physiology of the Global Change Research Institute of the Czech Academy of Sciences (Brno, Czech Republic) for stable carbon and nitrogen isotope composition (δ^13^C and δ^15^N).

Prior to analysis, the grain samples were homogenized using a Retsch MM400 ball mill (Retsch, Haan, Germany). Approximately 1.5 mg of the homogenized material was placed into tin capsules (Elementar Analysensysteme, Langenselbold, Germany) for isotopic analysis. Carbon (δ^13^C; ^13^C/^12^C) and nitrogen (δ^15^N; ^15^N/^14^N) isotope ratios were determined using a varioPYRO cube elemental analyzer (Elementar Analysensysteme, Langenselbold, Germany) coupled to an IsoPrime100 continuous-flow isotope ratio mass spectrometer (Isoprime, Manchester, UK). The elemental analyzer was operated in combustion mode at 960 °C.

The instrument stability and linearity across the expected ion current range obtained from the test samples were verified before each analytical session. The measurement accuracy, which was assessed through internal reference material (homogenized rye grain powder), presented standard deviations of < 0.05‰ for δ^13^C and < 0.15‰ for δ^15^N. The isotopic system was calibrated using a two-point calibration curve based on certified standard reference materials from the International Atomic Energy Agency (IAEA) and the United States Geological Survey (USGS): IAEA-600 (caffeine), USGS-24 (graphite), and USGS-32 (potassium nitrate).

The resulting δ^13^C and δ^15^N values are expressed in parts per thousand (‰) relative to the Vienna Pee Dee Belemnite (VPDB) and Atmospheric N_2_ (AIR) standards, respectively. These values were calculated using the standard formula, as follows:

1 $$\:{\delta\:}^{n}X=\left(\frac{{R}_{sample}}{{R}_{standard}}-1\right)\times\:1000$$

where *R* represents the ratio of the heavy isotope to the light isotope of element *X*.

### Drought resistance index (DRI) and aridity index (AI)

The drought resistance index (*DRI*) was calculated based on the formula developed by Lan^25^, as applied by Bennani et al.^26^, for each variety tested (Supplementary Table S1). The equation was as follows:

2 $$\:DRI=\frac{{Y}_{s}\times\:\left(\frac{{Y}_{s}}{{Y}_{p}}\right)}{mean\:{Y}_{s}},$$

where *Ys* and *Yp* represent the grain yield of drought-stressed and control plants, respectively, and the *mean Ys* is the mean grain yield of all varieties under drought stress.

The aridity index (*AI*) was calculated for each country of origin to quantify the degree of climate dryness of the respective growing regions in accordance with the method used by Bannayan et al.^27 ^(Supplementary Table S1). First, the Harvested Area and Yield for 175 Crops dataset by Monfreda et al.^28^ was used to identify key rye production areas in each country. This dataset was refined using national statistical data, varietal testing records, and national agronomic reports. The final dataset represents the most recent rye production areas for each country (Supplementary Table S3). Precipitation (*P*) and variables needed for calculations of potential evapotranspiration (*PET*) were retrieved from the ERA-5 Land database^29^. *PET* was subsequently estimated using the FAO-56 Penman-Monteith method^30^. These values were averaged across the established rye production areas for each country, covering the mean period from sowing to harvest^31^ over the climatic normal period 1991–2020. The AI was then calculated as the ratio of *P/PET* following UNEP^32^, and the mean AI for 1991–2020 was determined for each location of each country (Supplementary Table S1, S3).

### Data processing and statistical analyses

The Shapiro‒Wilk normality test and Brown‒Forsythe homogeneity of variance test were performed on all datasets using SigmaPlot 14.5 (Systat Software Inc., San Jose, California, USA; www.systat.com). If the data did not pass the Shapiro‒Wilk normality test, Box‒Cox transformation was applied using Statistica 14.0.0.15 software (TIBCO Software Inc., Palo Alto, California, USA; www.tibco.com) prior to ANOVA. Two-way ANOVA (*n* ≥ 4) was performed with the factors variety (V), treatment (T) and their interaction (V × T), followed by Tukey’s honest significant difference (*HSD*) test for unequal sample sizes^33^ for two significance levels (*p* = 0.01 and *p =* 0.05), to identify statistically significant differences between the rye varieties and treatments (control and drought stress) and to examine their interactive effects on grain weight per spike (GW), grain number per spike (GN), thousand-grain weight (TGW), harvest index (HI), straw and leaves weight (SLW), aboveground biomass per plant (AB), chlorophyll index (CI_F), and δ^13^C and δ^15^N values in grains. These analyses were performed using Statistica 14.0.0.15 software.

Paired *t* tests were performed via Origin software (OriginLab Corporation, Northampton, USA; https://www.originlab.com/) to identify whether the drought stress effects were statistically significant within traditional (*n* = 29 in the control group and *n* = 30 in the drought stress treatments) and modern (*n* = 69 in the control group and *n* = 70 in the drought stress treatments) variety groups (at two significance levels: *p* = 0.05 and *p* = 0.01).

The relative changes in the drought stress treatments compared with the control treatments (*RCC*) for each of these experimental traits were calculated using the following equation:

3 $$\:RCC=\frac{{AVG}_{c}-D}{{AVG}_{c}}\times\:100\%,$$

where *AVGc* is an arithmetic mean of a specific trait under the control treatment and *D* is a corresponding value under the drought stress treatment for a given variety and replication.

PCA was performed on a set of twelve quantitative traits (RY – year of variety registration based on Supplementary Table S1, AI, DRI, GW, GN, SLW, TGW, HI, AB, CI_F, δ^15^N and δ^13^C). All traits were transformed to Z‑scores by centering each variable to its mean and scaling it to unit variance, making the procedure equivalent to conducting PCA on the correlation matrix of the traits. The standardized matrix was then decomposed using singular value decomposition as implemented in scikit‑learn in Python, yielding component loadings and sample scores for each genotype. Group boundaries for modern and traditional genotypes were visualized as classical polygons defined by the convex hulls of individual observation scores, while genotype names were plotted at the mean position of their observations. A polar heatmap with cluster analysis for RCC, AI, DRI, and RY and correlation analysis (Pearson’s correlation coefficient matrix; *p* = 0.05) for the same datasets were generated via Origin software (OriginLab Corporation, Northampton, USA).

## Results

### Chlorophyll indices, yield traits, and grain δ^15^N and δ^13^C

**Fig. 2:**
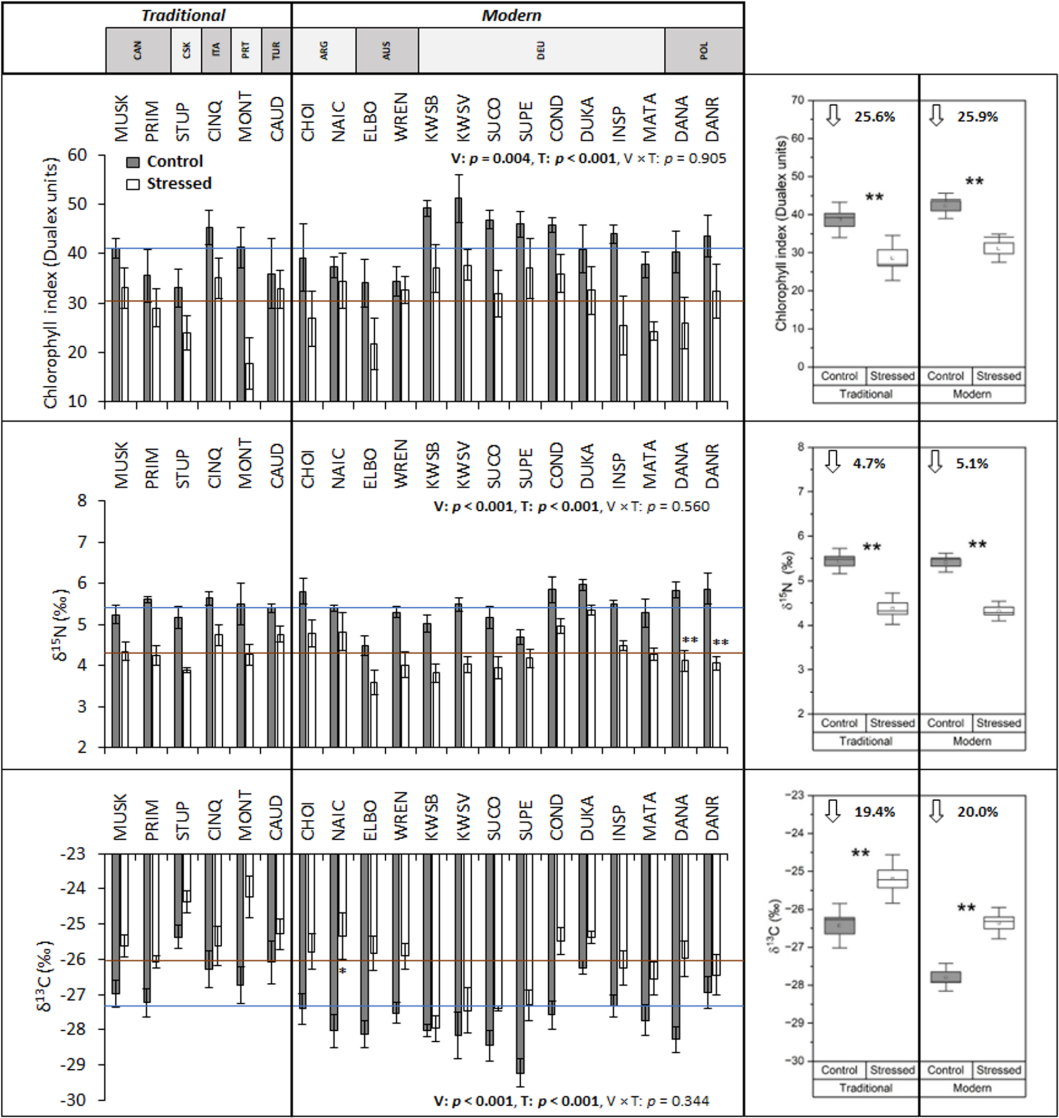
Mean values (columns) and standard errors of the means (error bars) for the chlorophyll index of a flag leaf, grain nitrogen isotope composition (δ^15^N), and grain carbon isotope composition in grains (δ^13^C) under control and drought stress treatments for individual winter rye varieties (left). The varieties are divided into traditional and modern varieties and by country of origin (see Supplementary Table S1 for country codes and full variety names). Statistically significant differences (*n* ≥ 4) among the control and drought stress (stressed) treatments within individual rye varieties according to two-way ANOVA with Tukey’s *HSD* test are denoted with * and ** symbols at significance levels of *p* = 0.05 and *p* = 0.01, respectively, if any differences are observed, and the effects of factor variety (V), treatment (T) and their interactive effects (V × T) are also shown. The Tukey *HSD* test results (letters) for *p* = 0.05 are presented in a separate Supplementary Table S4. The blue lines represent the total mean values of the control treatments, and the dark orange lines represent the total mean values of the drought stress treatments. Boxplot graphs (*n* ≥ 29) show the mean values ± 1 standard error of the mean (boxes), mean values ± 95% confidence interval (error bars), medians (horizontal lines within boxes), and mean values (squares within boxes) for the same traits presented in the bar charts (right). The * symbols and ** symbols denote statistically significant differences between the control and drought stress (stressed) treatments within traditional and modern varieties, respectively, according to paired *t* tests at significance levels of *p* = 0.05 and *p* = 0.01, respectively; *n.s.* denotes a statistically insignificant difference. The white arrows with numerical values represent the mean relative change in drought stress compared with the control treatment (RCC) for a specific trait within traditional and modern varieties (a downward/upward arrow indicates a decline/increase in the RCC, respectively).

Phenological observations during cultivation indicated small but consistent differences in developmental timing among varieties. Relative to the medium-maturing group (Stupicke S II, Prima, Musketeer, Naico, Cinquecento, Matador, Conduct, SU Performer, Inspector, SU Cossani, Dukato, Montalegre), phenological shifts among varieties were observed with deviations from − 4 days (Dankowskie Amber, Choigue, Elbon Gator 17, Wrens‑5) to + 2 ± 1 days (Caudar, KWS Binntto, KWS Vinetto, Dankowskie Rubin) (Supplementary Table S1). Drought-induced leaf senescence was assessed by measuring the chlorophyll index of the flag leaf (CI_F) at the end of the drought treatment in control and drought-stressed plants (Fig. 2). Both traditional and modern varieties presented similar drought-induced decreases in the mean CI_F (− 25.6% and − 25.9%, respectively). However, generally, the more modern the variety was, the greater the CI_F was under well-watered conditions, which indicates the higher productivity of modern varieties under optimal water availability. A similar trend was also observed for the above-ground biomass per plant (AB) and harvest index (HI).

**Fig. 3:**
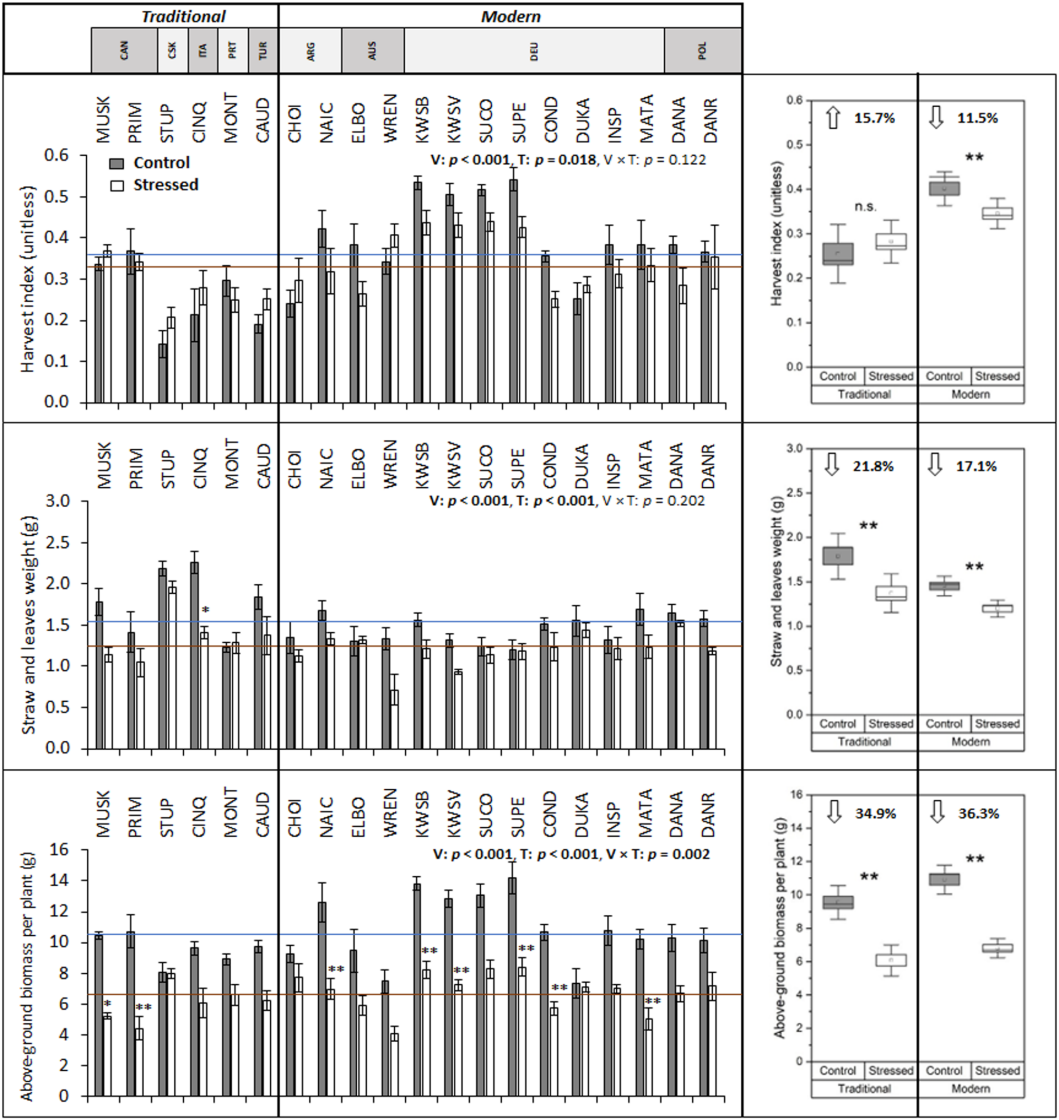
Mean values (columns) and standard errors of the means (error bars) for the harvest index, straw and leaf weights, and aboveground biomass per plant under the control and drought stress treatments for individual winter rye varieties (left). The varieties are divided into traditional and modern varieties and by country of origin (see Supplementary Table S1 for country codes and full variety names). Statistically significant differences (*n* ≥ 4) among the control and drought stress (stressed) treatments within individual rye varieties according to two-way ANOVA with Tukey’s *HSD* test are denoted with * and ** symbols at significance levels of *p* = 0.05 and *p* = 0.01, respectively, if any differences are observed, and the effects of factor variety (V), treatment (T) and their interactive effects (V × T) are also shown. The Tukey *HSD* test results (letters) for *p* = 0.05 are presented in a separate Supplementary Table S4. The blue lines represent the total mean values of the control treatments, and the dark orange lines represent the total mean values of the drought stress treatments. Boxplot graphs (*n* ≥ 29) show the mean values ± 1 standard error of the mean (boxes), mean values ± 95% confidence interval (error bars), medians (horizontal lines within boxes), and mean values (squares within boxes) for the same traits presented in the bar charts (right). The * symbols and ** symbols denote statistically significant differences between the control and drought stress (stressed) treatments within traditional and modern varieties, respectively, according to paired *t* tests at significance levels of *p* = 0.05 and *p* = 0.01, respectively; *n.s.* denotes a statistically insignificant difference. The white arrows with numerical values represent the mean relative change in drought stress compared with the control treatment (RCC) for a specific trait within traditional and modern varieties (a downward/upward arrow indicates a decline/increase in the RCC, respectively).

**Fig. 4:**
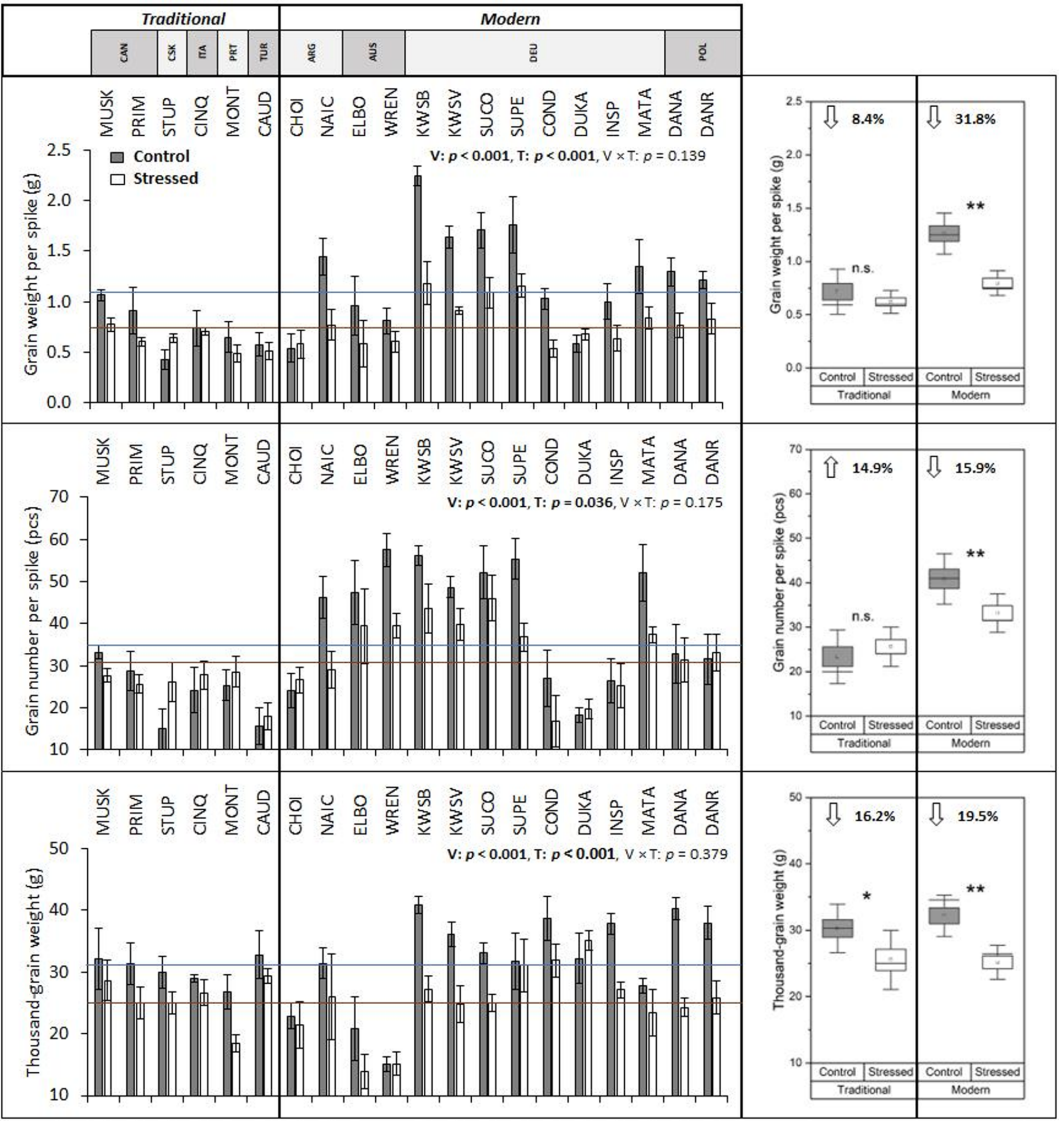
Mean values (columns) and standard errors of the means (error bars) for grain weight per spike, grain number per spike, and thousand-grain weight under control and drought stress treatments for individual winter rye varieties (left). The varieties are divided into traditional and modern varieties and divided based on country of origin (see Supplementary Table S1 for country codes and full variety names). Statistically significant differences (*n* ≥ 4) among the control and drought stress (stressed) treatments within individual rye varieties according to two-way ANOVA with Tukey’s *HSD* test are denoted with * and ** symbols at significance levels of *p* = 0.05 and *p* = 0.01, respectively, if any differences are observed, and the effects of factor variety (V), treatment (T) and their interactive effects (V × T) are also shown. The Tukey *HSD* test results (letters) for *p* = 0.05 are presented in a separate Supplementary Table S4. The blue lines represent the total mean values of the control treatments, and the dark orange lines represent the total mean values of the drought stress treatments. Boxplot graphs (*n* ≥ 29) show the mean values ± 1 standard error of the mean (boxes), mean values ± 95% confidence interval (error bars), medians (horizontal lines within boxes), and mean values (squares within boxes) for the same traits presented in the bar charts (right). The * symbols and ** symbols denote statistically significant differences between the control and drought stress treatments within traditional and modern varieties, respectively, according to paired *t* tests at significance levels of *p* = 0.05 and *p* = 0.01, respectively; *n.s.* denotes a statistically insignificant difference. The white arrows with numerical values represent the mean relative change in drought stress compared with the control treatment (RCC) for a specific trait within traditional and modern varieties (a downward/upward arrow indicates a decline/increase in the RCC, respectively).

In contrast to CI_F, drought-stressed plants of the traditional varieties showed higher HI values than control plants (Fig. 3), with a mean relative increase of + 15.7%. This trait was the most stable in the traditional varieties, in which drought increased mean grain number per spike (GN) by + 14.9% and reduced grain weight per spike (GW) by only − 8.4%. Thus, these varieties maintained or exceeded their varietal mean in thousand-grain weight (TGW; Fig. 4). However, in general, traditional varieties showed lower GW, GN, HI, and AB values than modern varieties under both well-watered and drought-stressed conditions. In the modern varieties, GW was the second most strongly affected trait after AB, with a mean drought-induced reduction of − 31.8%. The mean drought-induced decrease in HI in the modern varieties was − 11.5%, making it the second least affected parameter after grain δ^13^C. The most pronounced drought-induced decreases in the HI were observed in the modern varieties Elbon Gator 17 (− 31.2%) and Conduct (− 29.4%), both of which also presented severe decreases in other yield parameters and the CI_F (the mean decreases in all the parameters were − 32.3% and − 33.5%, respectively), indicating the drought sensitivity of these varieties.

AB (Fig. 3) was the most drought-sensitive trait in 12 out of the 20 rye varieties tested. The mean drought-induced declines in AB were − 34.9% and − 36.3% in traditional and modern varieties, respectively. Mean AB was higher in modern than in traditional varieties, by 11.9% in the control treatment and 10.8% under drought stress. The effect of drought on AB was statistically significant for the traditional varieties Musketeer (*p* = 0.014) and Prima (*p* < 0.001), and for the modern varieties Naico (*p* = 0.001), KWS Binntto (*p* = 0.005), KWS Vinetto (*p* = 0.003), SU Performer (*p* = 0.003), Conduct (*p* = 0.008), and Matador (*p* = 0.003). The smallest drought-induced reductions in AB were observed in Stupicke S II (− 1.41%) and Dukato (− 3.6%). Stupicke S II, the oldest variety in this study, also showed the highest stability in the GN, GW, and HI, with drought-induced increases of + 74.7%, + 51.1%, and + 44.5%, respectively; TGW remained slightly above the varietal mean under drought stress. Dukato showed similar trends, with the highest stability observed in GW (+ 16.4%), HI (+ 13.2%), TGW (+ 9.0%), and GN (+ 7.6%). In contrast, the greatest decreases in AB were observed in the traditional variety Prima (− 58.8%) and in the modern variety Matador (− 50.6%), with corresponding reductions in GW (− 33.6% and − 37.6%, respectively). In Matador, drought stress also caused an accelerated decrease in CI_F (− 35.8%) and GN.

Drought stress resulted in consistent declines in grain δ^15^N values across both traditional and modern varieties, with mean decreases of − 4.7% and − 5.1%, respectively (Fig. 2). In contrast, the mean δ^13^C values in the control treatment were slightly higher in the traditional than in the modern varieties (Fig. 2), indicating higher water use efficiency under well-watered conditions. On average, drought caused an increase in δ^13^C values by − 19.4% in traditional varieties and by − 20.0% in modern varieties.

Across varieties, the magnitude of the drought-induced reduction in δ^15^N was associated with the decline in TGW, and δ^15^N patterns were also associated with CI_F in the multivariate analyses (Fig. 6, 7).

Two-way ANOVA confirmed the statistically significant effects of both variety and treatment across all tested traits; however, a significant interactive effect was detected only for the AB (*p* = 0.002). The results of Tukey’s post hoc tests, which examined homogeneous groups among individual treatments across specific rye varieties separately for each tested trait, are summarized in Supplementary Table S4, where different letters denote statistically significant differences at a significance level of *p* = 0.05.

### Principal component analysis of the response to drought

**Fig. 5:**
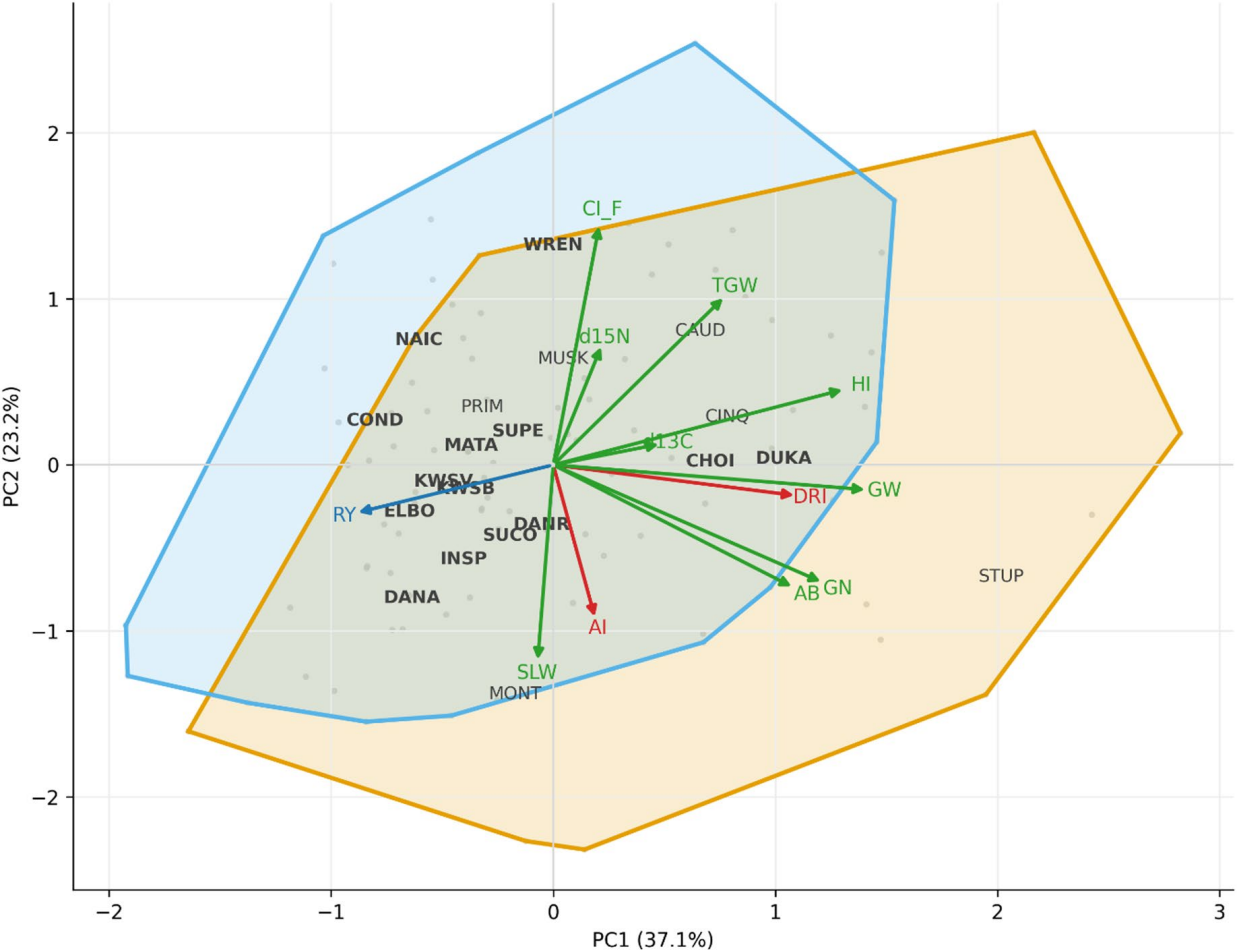
Principal component analysis (PCA) of relative changes in traits induced by drought stress compared with the control treatments for grain weight per spike (GW), grain number per spike (GN), straw and leaf weight (SLW), thousand-grain weight (TGW), harvest index (HI), aboveground biomass (AB), chlorophyll index (CI_F), isotopic composition (δ^13^C and δ^15^N) (displayed in green), year of variety registration (RY) (displayed in blue) and the climate indicators of the country of origin, aridity index (AI) and drought resistance index (DRI) (displayed in red). Arrows denote variable loadings. The modern rye varieties are indicated in bold, whereas the traditional varieties are presented in plain text. Points represent individual observations (gray). Genotype names are shown at the mean position of their replicate observations. Polygons represent group boundaries for modern and traditional genotypes, calculated as the convex hulls of observation scores (blue – modern varieties and orange – traditional varieties).

The PCA biplot (Fig. 5) shows separation between modern and traditional rye varieties along the first two principal components, which together explain 60.4% of the total variance (PC1 37.1% and PC2 23.3%). Modern varieties (blue polygon) occupy a slightly more compact region, indicating greater phenotypic uniformity, whereas traditional varieties (orange polygon) span a broader area, reflecting higher variability. Vectors representing relative reductions in key grain yield traits (GW, GN, and HI) under drought align strongly with PC1, are associated with positive PC1 scores and DRI, and are negatively associated with RY. This means that smaller negative reductions in these grain yield traits are associated positively with varieties originating from countries with higher DRI and with older, traditional varieties. The same vector direction, but with significantly lower loading than for yield traits, is evident for the change in δ^13^C values under drought. In addition to these features, the AB response to drought, which is very closely associated with the relative GN response, shows the same response direction as PC1. PC2 distinguishes genotypes mainly by drought response of traits such as CI_F and SLW, which are negatively associated with each other. In addition, SLW reduction by drought is positively associated with AI, and the reduction in CI_F is positively (but weaker) associated with changes in δ^15^N under drought. TGW’s response is positioned between PC1 and PC2 (CI_F and GW), but it also generally shows a negative association with RY. Among modern varieties, Choigue and Dukato showed greater similarity to traditional genotypes based on their positions on PC1 and PC2, whereas among traditional varieties, Prima in particular showed greater similarity to modern varieties in its response to drought. The Montalegre and Wrens-5 varieties, which are opposite each other on PC2, show a close positive association with the response of the CI_F (Wrens-5) and SLW (Montalegre) traits to drought and vice versa (Fig. 5).

### Polar heatmap with cluster analysis of the response to drought

**Fig. 6:**
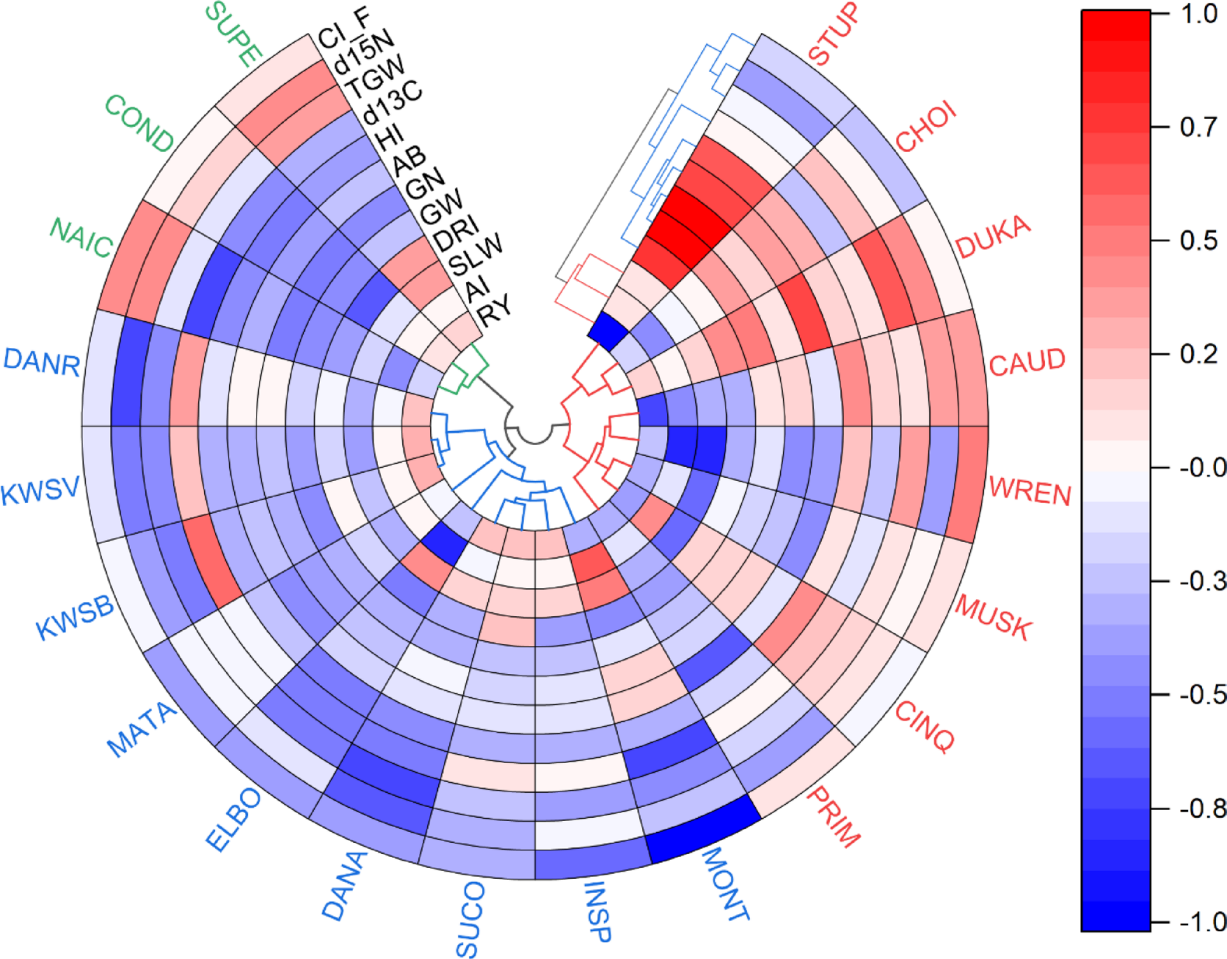
Polar heatmap with cluster analysis for relative changes in the drought stress treatments compared with the control treatments for grain weight per spike (GW), grain number per spike (GN), straw and leaf weight (SLW), thousand-grain weight (TGW), harvest index (HI), aboveground biomass (AB), chlorophyll index (CI_F), isotopes δ^13^C (d13C) and δ^15^N (d15N), and year of variety registration (RY), aridity index (AI), and drought resistance index (DRI).

The polar heatmap (Fig. 6) clustered varieties according to the relative drought response of individual traits, indicators of origin (AI and DRI) and year of registration (RY). Within the polar heatmap, three main clusters were identified. The cluster of varieties marked in red mainly represents traditional varieties, with two modern varieties (Choigue and Dukato) that are very similar to traditional varieties in terms of their response to drought. However, even within this group, there are clear differences in the response profile of individual traits. Some varieties, such as Stupicke S II, Choigue, Dukato, maintained high relative yields (GW) under drought stress, whereas others, such as Musketeer, Prima, and Wrens-5, showed higher GW sensitivity to drought. This cluster was generally characterized by a stable HI and a partially stable TGW under drought conditions, with relative δ^13^C values hovering around the mean.

The cluster indicated by blue color included mostly modern rye varieties that presented rather high sensitivity of most yield traits such as GW, GN, TGW and HI to drought similar to CI_F, and δ^15^N. This group, on the other hand, was characterized by rather low sensitivity of the SLW trait to drought. Some varieties, particularly KWS Binntto, KWS Vinetto, SU Cossani, and Dankowskie Rubin, presented relatively high δ^13^C values.

The cluster of genotypes indicated by green color included modern varieties (Naico, Conduct, and SU Performer) with high sensitivity of GW, GN, AB, and HI to drought stress, but relatively good tolerance in CI_F and TGW. In addition, this group of varieties showed only a slight reduction in δ^15^N and, conversely, a more pronounced decrease in δ^13^C (more negative values) in response to drought.

### Correlation analysis of the response to drought

**Fig. 7:**
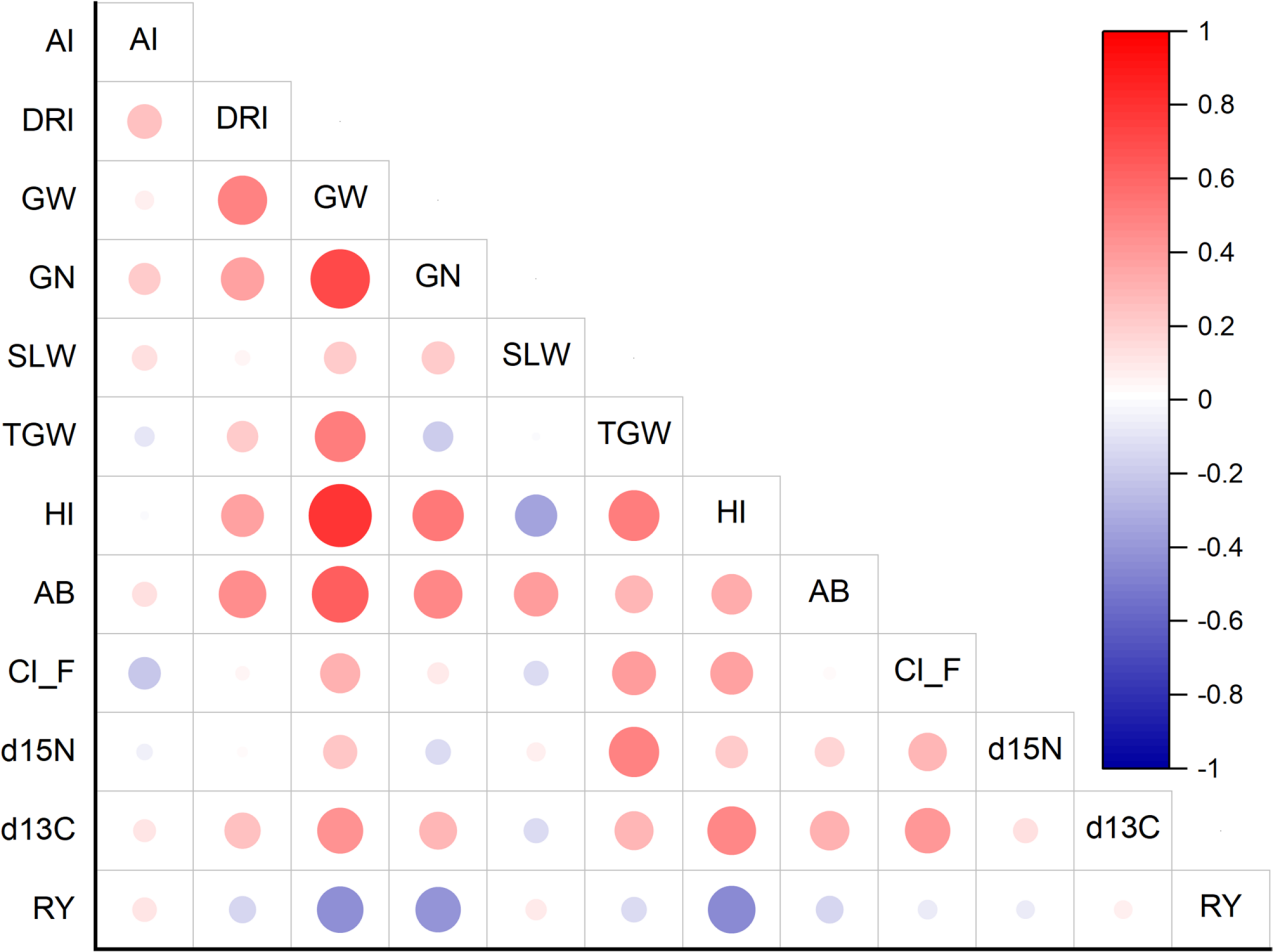
Pearson’s correlation coefficient (*p* = 0.05) matrix for relative changes in the drought stress treatments compared with the control treatments for grain weight per spike (GW), grain number per spike (GN), straw and leaves weight (SLW), thousand-grain weight (TGW), harvest index (HI), aboveground biomass (AB), chlorophyll index (CI_F), isotopes δ^13^C (d13C) and δ^15^N (d15N), and year of variety registration (RY), aridity index (AI), and drought resistance index (DRI).

The results of the correlation analysis (Fig. 7) revealed strong associations among production parameters, especially between the GW, GN, HI, and AB. Specifically, the decrease in AB due to drought was closely associated with decreases in GW and GN, highlighting that yield losses were mainly associated with lower GN. In contrast, the TGW was only weakly correlated with the AB, and the correlation of the TGW with the GW was weaker than the correlation of the GN with the GW.

Negative correlations were observed between RY and the relative response of yield parameters. This correlation was particularly strong for GW, GN, and HI, indicating that more recently registered varieties were more sensitive to drought, whereas older genotypes presented higher tolerance to drought. Furthermore, the drought-induced decrease in TGW was well indicated by the reduction in δ^15^N.

## Discussion

### Drought timing and yield component sensitivity

Rye is particularly susceptible to drought during the period of heading, anthesis and early grain filling. This period is when the number of grains per spike is most strongly affected, leading to significant yield reductions under drought stress^34^.

Grain yield in cereals is the product of grain number (GN) and mean grain weight (TGW), but these components differ markedly in developmental timing, drought sensitivity, and capacity for compensation. GN largely reflects spike fertility (floret survival and grain set) and is therefore highly sensitive to resource limitation during the rapid spike growth period immediately before and around anthesis^35^. Analyses in wheat show that GN is tightly linked to conditions in the 30 days preceding anthesis, representing the spike growth period, which controls spike dry‑weight accumulation and floret survival^36^. In contrast, TGW is more closely related to grain filling and to the potential size of individual grains, the latter being partially determined before anthesis but strongly influenced by post‑anthesis assimilate supply^37^. Under moderate stress, decreases in GN can be partly compensated by stable or increased TGW because fewer grains reduce competition for assimilates, allowing remaining grains to better realize their potential size. However, such compensation is usually incomplete when drought also restricts post‑anthesis photosynthesis and accelerates senescence^35^. A key buffering mechanism is the mobilization of pre‑anthesis stem reserves. The contribution of remobilized assimilates to grain growth increases markedly under stress, while high stem water‑soluble carbohydrate storage and efficient remobilization have been repeatedly linked to maintaining kernel weight under post‑anthesis water limitation^37,38^. In water‑limited or terminal‑drought environments, water‑soluble carbohydrates may contribute up to 40–70% of final grain weight, making this mechanism crucial when current photosynthesis collapses^38^.

In this study, the drought-sensitive period was extended to include approximately one week before the beginning of the sensitive period and after its end. Drought before anthesis mainly reduces GN, which is crucial yield component set before anthesis, in contrast to the TGW, which is formed later^34^. Although drought corresponding to soil moisture just above the wilting point was induced in our study, several varieties showed relatively small responses of yield components to drought (although in other varieties the reduction exceeded 40%), which is probably due to the early onset of drought induction in the second half of stem elongation and later possibility of drought impact compensation after drought termination. However, this early drought is of growing importance in Central Europe in connection with the ongoing climate change. Recent studies demonstrate that droughts in Central Europe are increasingly shifted toward earlier parts of the growing season, with a marked rise in April–June flash droughts^39^. Moreover, climate‑change‑driven soil‑moisture depletion occurs well before summer, effectively advancing the onset of physiological water stress for crops^40^. This earlier drought timing can suppress stem and vegetative development disproportionately, allowing some genotypes to acclimate or compensate during grain filling, which explains why yield‑component reductions were moderate in some varieties despite severe overall stress.

### Variety-specific drought resistance and contrasting response strategies

The findings of this study highlight that drought resistance in rye is associated primarily with RY. Older varieties generally present a low relative GW reductions similar to individual yield components under drought conditions, despite their relatively low yield potential. The heatmap combined with the cluster analysis highlighted distinct drought response strategies among rye varieties, with older varieties generally exhibiting greater stability in terms of relative yield parameters and some modern varieties showing adaptation via improved water use efficiency (δ^13^C) or biomass maintenance (δ^15^N).

Varieties Stupicke S II, Choigue, and Caudar displayed a GN‑stable strategy, maintaining grain number (GN) under drought with only minor reductions. This indicates that these varieties were more effective at preserving spike growth and floret survival during the pre‑anthesis sensitive period, a mechanism consistent with conserved reproductive physiology in cereals during stem elongation and floret differentiation^41^. On the other hand, varieties Dukato, Wrens-5, Musketeer, Cinquecento, Choigue, and Caudar exhibited a TGW‑stable strategy, maintaining or even slightly increasing thousand‑grain weight (TGW) under drought. This pattern suggests a stronger reliance on sustained assimilate supply to grains and/or more efficient remobilization of pre‑anthesis stem carbohydrates during grain filling mechanisms repeatedly identified as key buffering strategies under water limitation^42^.

These contrasting GN‑stable versus TGW‑stable strategies can be interpreted as differences in whether drought tolerance is expressed mainly through maintaining sink size at flowering, that is, preserving floret survival and grain set during the pre‑anthesis critical period^35^, or through protecting grain filling via sustained assimilate supply and/or stronger remobilization of stem reserves^37,38^. Because remobilization contributions rise under stress, genotypes that better store and mobilize stem carbohydrates can maintain TGW even when current photosynthesis is constrained^37,38^, whereas genotypes that preserve GN likely maintain spike growth and fertility during the pre‑anthesis spike growth period, when assimilate shortage most strongly reduces floret survival^36,35^.

### Biomass and harvest index responses under drought

The results of our study showed that HI changes fairly little as a result of drought, and relatively less than GW or AB. This is particularly evident in traditional varieties, where HI even increased slightly in some varieties due to drought, but even in the case of modern varieties, the changes in HI were relatively small. Although harvest index (HI) typically decreases under reproductive‑stage drought, its response strongly depends on the timing of stress. A large meta‑analysis of drought in cereals shows that the growth stage strongly modifies drought effects, with reproductive‑stage drought reducing yield and HI most strongly, whereas earlier drought can affect biomass more than grain yield (i.e., lowering the denominator of HI)^43^. This pattern was consistent with our observations of markedly reduced above‑ground biomass and accelerated senescence (lower CI_F), indicating that the stress was both early and physiologically severe. The literature also shows that stem carbohydrate mobilization can buffer grain filling under drought, and that genotypes differ widely in their ability to remobilize stem reserves^44^. Genotypes with inherently lower HI under control conditions often have greater reserve mobilization capacity, enabling them to maintain or even increase HI when stress reduces vegetative growth early.

Under drought conditions, rye plants increasingly rely on pre-anthesis carbohydrate reserves for grain filling, thereby mitigating reductions in GW. This remobilization is crucial for maintaining yield under water-limited conditions^34^. Varieties with lower drought-induced senescence and prolonged green area retention (in this study, e.g., SU Performer, Caudar, Wrens-5, or Naico) are generally characterized by lower sensitivity of the TGW to drought. However, the ability to accumulate assimilates before flowering also plays an important role, which differentiates the relationship between the effects of drought on the CI_F and TGW values. Thus, while RY is the unifying factor in drought resistance, considerable diversity exists in the traits contributing to resistance. Given their lower yield potential, older varieties are better suited as donors of drought resistance traits than as direct replacements for modern rye varieties.

A significant variety × treatment interaction was detected only for AB, indicating that rye varieties differed mainly in their vegetative growth responses to drought, while their reproductive processes responded more uniformly across genotypes. AB is inherently more plastic and closely tied to vegetative vigor and early canopy development, which can differ substantially among varieties even before drought onset. In contrast, reproductive traits such as GN, TGW, and HI were affected by drought in a largely parallel manner across varieties, consistent with the broadly conserved reproductive response of cereals during the sensitive stem‑elongation to pre‑anthesis period, when assimilate limitation reduces spike growth and floret survival similarly across genotypes^42,41^.

High-biomass genotypes (typical for some modern genotypes) often exhibit greater sensitivity to drought because their large canopy and stem mass create a higher transpirational demand, leading to faster soil moisture depletion and earlier onset of physiological stress. Recent studies across cereals show that genotypes with vigorous biomass production tend to suffer larger reductions in photosynthetic performance, water status, and yield components when water becomes limiting^45^. Moreover, high biomass can reduce drought resilience by increasing dependence on post-anthesis assimilate supply, whereas genotypes with more moderate biomass often possess relatively stronger stem reserve mobilization or maintain more conservative growth strategies under stress^46^. Recent findings from Findurová et al.^47^ further support this interpretation, showing that overly vigorous early biomass accumulation is associated with sharper declines in physiological performance and yield stability under drought, underscoring the negative role of excessive vegetative growth when water becomes limiting. In this study, drought progression was standardized by daily weighing and adding small amounts of water so that all drought-treated pots tracked the same soil-moisture trajectory (using the second-wettest pot as the daily reference), thereby minimizing potential differences in drying rate among genotypes due to variation in biomass. Nonetheless, this approach may help to clarify why certain lower-yielding genotypes exhibited less relative yield loss.

The superior drought resistance of older cereal crop varieties compared with modern high-yield varieties is often attributed to a combination of traits, including more developed root system, tighter stomatal regulation, stronger osmotic adjustment, and enhanced antioxidant defenses^48–50^. Genetic studies have identified unique alleles in older varieties linked to root traits, emphasizing the role of these alleles in drought resilience^51,52^. Older varieties frequently exhibit deeper roots, greater lateral root length, higher root-to-shoot ratios, and increased xylem vessel adaptations, increasing water uptake under drought^48,53^. In older varieties, tighter (often ABA-mediated) stomatal control and, in some cases, reduced stomatal density can limit transpirational water loss while maintaining comparatively higher CO_2_ assimilation per unit stomatal conductance (i.e., higher intrinsic WUE) under drought stress^54,55^. Compared with modern varieties, some older varieties excel in osmotic adjustment through increased proline and soluble sugar accumulation, increased ROS scavenging, reduced oxidative damage, and improved chlorophyll retention under stress^50^. Older varieties achieve a better balance between root growth and grain filling, thus buffering against the impact of drought, whereas modern varieties prioritize yield-related traits, such as optimized reproductive biomass, at the expense of vegetative resilience, thus reducing their adaptability under stress^56,50^.

### Physiological and isotopic proxies for screening drought tolerance

In the present study, we did not directly quantify root architecture or instantaneous leaf gas exchange (A, gs) because our experimental design prioritized a high number of varieties with a proper number of replications and relied, therefore, on high-throughput measurements suitable for screening under controlled drought. Instead, we used CI_F and grain δ^13^C/δ^15^N as integrative proxies capturing drought effects on senescence, intrinsic WUE and nitrogen dynamics over the stress period. Future work should combine these screening indicators with targeted root phenotyping and leaf gas-exchange measurements to resolve the underlying mechanisms.

Stable isotope analysis of grain δ^13^C and δ^15^N provides time-integrated indicators of plant water and nitrogen relations during the period when assimilates supporting grain filling are formed. In C3 cereals, less negative (higher) grain δ^13^C value generally reflects lower Ci/Ca, typically driven by reduced stomatal conductance relative to photosynthetic capacity (iWUE = A/gs;^57^, and is therefore widely used as an integrative proxy for intrinsic WUE. Consistent with previous studies on wheat^58,59^, barley^60^, and triticale^61^, we observed higher (less negative) grain δ^13^C values in drought-treated rye varieties, indicating improved intrinsic WUE under water limitation. However, the relationship between δ^13^C and WUE can be modified by environmental conditions (e.g., irradiation, soil texture, irrigation regime), and higher intrinsic WUE may come at the cost of reduced carbon assimilation and growth, which can limit yield potential under non-drought conditions^62,63^. Grain δ^15^N reflects both the isotopic signature of plant-available N and net fractionations associated with N uptake, assimilation, and within-plant redistribution. Accordingly, treatment- or genotype-driven shifts in grain δ^15^N can be interpreted as integrative indicators of changes in N acquisition versus internal remobilization and allocation to grains under drought^64^.

While previous studies have validated δ^13^C as a drought-related proxy in rye, few studies have combined δ^13^C and δ^15^N to assess drought-related traits. In particular, δ^13^C has been analyzed in grains and flag leaves to evaluate its relationship with WUE and drought adaptability under controlled water regimes, revealing correlations with grain yield under severe drought but inconsistencies across years and tissue types^65^. In durum wheat, integrating δ^13^C (WUE) and δ^15^N (nitrogen use efficiency – NUE) distinguished genotypic drought tolerance, with higher δ^13^C values reflecting water stress adaptation and δ^15^N values reflecting shifts in nitrogen metabolism and internal nitrogen recycling^66,67^. In accordance with these studies, this study revealed lower δ^15^N values in the grains of drought-treated plants than in those of well-watered plants.

Several mechanisms may contribute to the observed decline in δ^15^N values under drought stress, reflecting shifts in the balance between nitrogen demand and supply. Because all varieties received the same N source and growth substrate, treatment- and genotype-related differences in grain δ^15^N primarily reflect net fractionation and within-plant N partitioning during grain filling. Reduced nitrogen requirements for growth and biomass production in drought-stressed plants lead to increased isotopic fractionation against ^15^N during inorganic N uptake and assimilation, particularly for NH_4_^+^, resulting in lower δ^15^N values in plant tissues^64^. In addition, drought may reduce post-anthesis N uptake and increase reliance on internally remobilized N for grain filling, which can further decrease grain δ^15^N values^68^. Finally, drought-induced reductions in stomatal conductance and transpiration may suppress gaseous N losses (e.g., NH_3_ volatilization), processes that preferentially remove ^14^N, thus contributing to the observed decline in grain δ^15^N values^69,70^. In our dataset, the decrease in δ^15^N co-varied with reduced TGW and lower CI_F, consistent with the interpretation that varieties showing stronger δ^15^N declines experienced a greater limitation in N delivery to grains and a faster progression of drought-induced senescence.

### Implications for breeding and practical selection

From a breeding perspective, our results suggest that improving drought tolerance in rye without sacrificing productivity will require combining high yield potential of modern varieties with specific drought-stability traits identified here. Traditional varieties that showed low relative yield loss and stable yield components under drought represent valuable donor parents, particularly for traits linked to maintaining sink size (GN-stable strategy; e.g., Stupicke S II, Choigue, Caudar) or sustaining grain filling (TGW-stable strategy; e.g., Dukato, Wrens-5, Musketeer, Cinquecento, Naico). In practical breeding programs, these contrasting strategies can be introgressed into high-yield genetic backgrounds and selected under managed early-season drought scenarios similar to those increasingly observed in Central Europe. Importantly, the integrative traits used in this study (RY, CI_F, and grain δ^13^C/δ^15^N) provide a feasible high-throughput screening toolbox to identify progeny that maintain yield stability while retaining high absolute yield potential.

## Conclusion

Winter rye constitutes one of the alternative cereal crops to winter wheat, with the potential for higher drought resilience. This study assessed the drought responses of twenty winter rye varieties differing in year of registration and geographic origin, with water limitation imposed during the sensitive stages from stem elongation through anthesis and early grain filling. Although the varieties differed in yield potential, their responses to drought followed consistent patterns.

Modern varieties maintained higher absolute yields under both well‑watered and drought conditions, reflecting their greater inherent productivity. However, they also showed larger proportional (relative) reductions in grain yield and associated traits, indicating a higher sensitivity to drought stress. In contrast, traditional varieties, despite their lower yield potential, displayed greater relative stability of key yield components, particularly grain number (GN) and thousand‑grain weight (TGW).

The analysis of individual drought‑tolerance strategies revealed that some traditional varieties (e.g., Stupicke S II, Choigue) and selected modern ones (e.g., Caudar) maintained GN under drought, whereas other varieties, including both modern (Dukato, Wrens‑5) and traditional (Musketeer, Cinquecento), showed greater TGW stability. A few varieties combined both mechanisms. These contrasting responses highlight that rye drought tolerance arises from multiple pathways, involving either preservation of sink size around anthesis or protection of grain filling via assimilate supply and remobilization.

Correlation analysis further indicated that drought sensitivity was associated with above‑ground biomass responses, with high‑biomass varieties exhibiting stronger relative reductions in both AB and GW. Additionally, the drought-induced decrease in TGW was associated with a reduction in δ^15^N.

Overall, these findings demonstrate that while modern varieties remain superior in terms of absolute grain production, traditional varieties offer valuable genetic resources for enhancing drought resilience, particularly for maintaining the stability of yield components under early‑season water deficit. Their traits should therefore be considered for incorporation into future breeding programs rather than as direct replacements for modern high‑yielding varieties in commercial production.

## Supplementary Information

The Supplementary Information provides additional information on the rye varieties used (Supplementary Table S1), applications of fertilizers and treatments for pests and diseases (Supplementary Table S2), input data for the aridity index calculation (Supplementary Table S3), the results of Tukey’s *HSD* test of two-way ANOVA presented in Figs. 2, 3 and 4 (Supplementary Table S4) and the courses of meteorological variables recorded in the vegetation hall of Mendel University in Brno and in the greenhouse of PSI Drásov (Supplementary Fig. S1, S2).

Below is the link to the electronic supplementary material.

Supplementary Material 1

## Data Availability

The authors confirm that the data supporting the findings of this study are available within the article and its Supplementary Information.The Authors’ Original Manuscript was uploaded to the ZENODO preprint server: Hlaváčová M, Klem K, Pytela J, Urban O, Pernicová N, Balek J, Semerádová D, Fischer M, Rötter RP, Appiah M, Hlavinka P, Horáková V, Škarpa P, Trnka M (2025) Traditional rye varieties exhibit drought tolerance traits but maintain lower yields than modern varieties under drought stress. Zenodo. https://doi.org/10.5281/zenodo.17599250.
