# Supplementary material for: Traditional rye varieties exhibit drought tolerance traits but maintain lower yields than modern varieties under drought stress
Source: Sci Rep. 2026 May 5;16:20650. doi: 10.1038/s41598-026-51544-5 (PMC13333828; doi:10.1038/s41598-026-51544-5)
Supplement: Supplementary file 1 — Supplementary Material 1 [file 41598_2026_51544_MOESM1_ESM.docx]

**SupplementaRY INFORMATION**

**Traditional rye varieties exhibit drought tolerance traits but maintain lower yields than modern varieties under drought stress**

Marcela Hlaváčová^1,2*^, Karel Klem^2,3^, Jaromír Pytela^4^, Otmar Urban^3^, Natálie Pernicová^3^, Jan Balek^1,2^, Daniela Semerádová^1,2^, Milan Fischer^1,2,5^, Reimund P. Rötter^6^, Mercy Appiah^6^, Petr Hlavinka^1,2^, Vladimíra Horáková^7^, Petr Škarpa^8^ and Miroslav Trnka^1,2^

^1^ Department of Climate Change Impacts on Agroecosystems, Global Change Research Institute of the Czech Academy of Sciences, Bělidla 986/4a, Brno, 60300, Czech Republic

^2^ Department of Agrosystems and Bioclimatology, Faculty of AgriSciences, Mendel University in Brno, Zemědělská 1665/1, Brno, 61300, Czech Republic

^3^ Laboratory of Ecological Plant Physiology, Global Change Research Institute of the Czech Academy of Sciences, Bělidla 986/4a, Brno, 60300, Czech Republic

^4^ Plant Phenotyping and Biotechnology Platform, Photon Systems Instruments, Průmyslová 470, Drásov, 66424, Czech Republic

^5^ Department of Matters and Energy Fluxes, Global Change Research Institute of the Czech Academy of Sciences, Bělidla 986/4a, Brno, 60300, Czech Republic

^6^ Faculty of Agricultural Sciences, Department of Crop Sciences, Tropical Plant Production and Agricultural Systems Modelling, Georg-August-Universität Göttingen, Wilhelmsplatz 1, Göttingen, 37073, Germany

^7^ Plant Production Section, National Plant Variety Office, Department of Utility Value Testing, Central Institute for Supervising and Testing in Agriculture, Hroznová 63/2, Brno, 60300, Czech Republic

^8^ Department of Agrochemistry, Soil Science, Microbiology and Plant Nutrition, Faculty of AgriSciences, Mendel University in Brno, Zemědělská 1665/1, Brno, 61300, Czech Republic

***Corresponding author**

Marcela Hlaváčová, Department of Climate Change Impacts on Agroecosystems, Global Change Research Institute of the Czech Academy of Sciences, Bělidla 986/4a, 603 00 Brno, Czech Republic.

E-mail: [hlavacova.m@czechglobe.cz](file:///F:\Prace\Konference_publikace-2025\Clanek-zito%20ozime%202019\Postup%20reseni\FINALNI_VERZE_CLANKU\PO%20REVIZI%20PRAVOPISU\Scientific_Reports\REVISED_VERSION-II.ROUND\hlavacova.m@czechglobe.cz)

Supplementary Information, containing nine pages, consists of four tables (Supplementary Table S1, S2, S3, and S4) and two figures (Supplementary Fig. S1 and S2).

**Supplementary Table S1**

Winter rye varieties tested in this study, including variety abbreviation, countries of origin, years of seed registration in the GRIN Czech plant genebank (<https://grinczech.vurv.cz/gringlobal/>), variety type (traditional or modern), registration status in the National List of Varieties of the country of origin (registered – R or not registered – NR), phenological deviation (days) relative to the medium-maturing group (medium-maturing = 0 days; negative values indicate earlier and positive values later development), and the aridity index (AI) and drought resistance index (DRI) for each variety.

| Variety | Abbreviation | Origin^1^ | Registration year^3^ | Variety type | Registration in the National List of Varieties | Phenological deviation (days) relative to the medium-maturing group | AI^4^ | DRI^5^ |
| --- | --- | --- | --- | --- | --- | --- | --- | --- |
|  |  |  |  |  |  |  |  |  |
| Stupicke S II | STUP | Czechoslovakia, CSK**^2^** | 1953 | Traditional | NR | 0 days | 1.232 | 1.302 |
| Caudar | CAUD | Turkey, TUR | 1967 | Traditional | NR | +2 days | 0.664 | 0.612 |
| Cinquecento | CINQ | Italy, ITA | 1985 | Traditional | NR | 0 days | 1.686 | 0.900 |
| Musketeer | MUSK | Canada, CAN | 1987 | Traditional | R | 0 days | 1.024 | 0.754 |
| Prima | PRIM | Canada, CAN | 1987 | Traditional | R | 0 days | 1.024 | 0.539 |
| Montalegre | MONT | Portugal, PRT | 1987 | Traditional | NR | 0 days | 1.902 | 0.494 |
| Elbon Gator 17 | ELBO | Australia, AUS | 1991 | Modern | NR | –4 days | 0.219 | 0.480 |
| Wrens-5 | WREN | Australia, AUS | 1991 | Modern | NR | –4 days | 0.219 | 0.606 |
| Choigue | CHOI | Argentina, ARG | 1995 | Modern | NR | –4 days | 0.641 | 0.830 |
| Naico | NAIC | Argentina, ARG | 1995 | Modern | NR | 0 days | 0.641 | 0.552 |
| Matador | MATA | Germany, DEU | 2002 | Modern | NR | 0 days | 1.168 | 0.705 |
| Conduct | COND | Germany, DEU | 2007 | Modern | R | 0 days | 1.168 | 0.368 |
| SU Performer | SUPE | Germany, DEU | 2013 | Modern | R | 0 days | 1.168 | 1.026 |
| Dukato | DUKA | Germany, DEU | 2013 | Modern | R | 0 days | 1.168 | 1.061 |
| Variety | Abbreviation | Origin^1^ | Registration year^3^ | Variety type | Registration in the National List of Varieties | Phenological deviation (days) relative to the medium-maturing group | AI^4^ | DRI^5^ |
| SU Cossani | SUCO | Germany, DEU | 2014 | Modern | NR | 0 days | 1.168 | 0.933 |
| Dankowskie Amber | DANA | Poland, POL | 2014 | Modern | R | –4 days | 1.111 | 0.603 |
| Dankowskie Rubin | DANR | Poland, POL | 2014 | Modern | R | ± 1 day | 1.111 | 0.766 |
| Inspector | INSP | Germany, DEU | 2016 | Modern | R | 0 days | 1.168 | 0.547 |
| KWS Binntto | KWSB | Germany, DEU | 2017 | Modern | R | +2 days | 1.168 | 0.838 |
| KWS Vinetto | KWSV | Germany, DEU | 2018 | Modern | R | +2 days | 1.168 | 0.675 |

^1^Country of variety origin was abbreviated according to standard ISO 3166: <https://www.iban.com/country-codes>

^2^No longer a country since 1 January 1993 (the Czech Republic and Slovak Republic emerged).

^3^Traditional (1953–1990) and modern (1991–2018) varieties based on the seed registration in the plant genebank of the GRIN Czech (<https://grinczech.vurv.cz/gringlobal/>) or year of registration to the National Varietal List of a specific country of a variety origin in the case of no data availability in the GRIN Czech database (SU Performer, KWS Binntto, SU Cossani and KWS Vinetto).

^4^AI – aridity index, *AI = P/PET* according to UNEP (1992), where *P* (mm) is precipitation and *PET* (mm) is potential evapotranspiration. AI was calculated for the period from sowing to harvest of rye (country-specific) as the mean AI for the most recent climate normal (1991–2020). Aridity indices were calculated only for the main rye-production regions in each country (based on recent production patterns up to 2023 and/or available literature sources; see Supplementary Table S3 for details.

^5^DRI – Drought resistance index by Lan (1998), as presented in Bennani et al. (2017), *DRI = [Ys × (Ys/Yp)]/mean Ys*, where *Ys* and *Yp* are the grain yields of a variety under stress and the control treatment, respectively, and the *mean Ys* is the mean grain yield of all varieties under stress treatment.

**Supplementary Table S2**

Dates and products used for fertilization and pest/disease control during the experiment, with applied dose/concentration and listed active ingredients.

| Application date | Substance (dose/concentration; product type) | Active ingredients |
| --- | --- | --- |
| 5 April 2019 | calcium ammonium nitrate (15.4 kg ha^-1^; *F*) | 27% N (13.5% N-NO_3_^-^ + 13.5% N-NH_4_^+^), 7% CaO, 5% MgO |
| 8 April 2019 | Boogie _Xpro_ (0.3%; *FC*)^1^ | 50 g L^-1^ bixafen, 100 g L^-1^ prothioconazole, 250 g L^-1^ spiroxamine |
| 8 April 2019 | NURELLE D (0.2%; *IC*)^1^ | 500 g L^-1^ chlorpyrifos, 50 g L^-1^ cypermethrin |
| 15 April 2019 | FERTILEADER Vital-954 (0.2%; *F*) | 104 g L^-1^ N, 58 g L^-1^ P_2_O_5_, 46 g L^-1^ K_2_O, 1160 mg Mn, 580 mg B, 580 mg Zn, 232 mg Cu, 232 mg Fe, 116 mg Mo, Seactive® (IPA, glycin betain, amino acids) |
| 30 April 2019 | calcium ammonium nitrate (15.4 kg ha^-1^; *F*) | 27% N (13.5% N-NO_3_^-^ + 13.5% N-NH_4_^+^), 7% CaO, 5% MgO |
| 30 April 2019 | Proteus ® 110 OD (0.2%; *IC*)^1^ | 100 g L^-1^ thiacloprid, 10 g L^-1^ deltamethrin |
| 30 April 2019 | Boogie _Xpro_ (0.3%; *FC*)^1^ | 50 g L^-1^ bixafen, 100 g L^-1^ prothioconazole, 250 g L^-1^ spiroxamine |
| 16 May 2019 | Proteus ® 110 OD (0.2%; *IC*)^1^ | 100 g L^-1^ thiacloprid, 10 g L^-1^ deltamethrin |
| 16 May 2019 | Boogie _Xpro_ (0.3%; *FC*)^1^ | 50 g L^-1^ bixafen, 100 g L^-1^ prothioconazole, 250 g L^-1^ spiroxamine |

Note: Abbreviations: *F*, fertilizer; *FC*, fungicide; *IC*, insecticide.

^1^Combined treatment at one dose.

**Supplementary Table S3**

Input data used to calculate the aridity index (AI) for each country of variety origin, including the main rye-producing region selected for the calculation, data sources, years used to define recent production areas, and the sowing/harvest dates (Julian days) used to derive country- specific mean precipitation (P; mm) and potential evapotranspiration (PET; mm) for the 1991–2020 climate normal.

| Country | Rye-production area used for AI | Data source | Website | Years used to define the recent production area | Sowing date (Julian days)^5^ | Harvest date (Julian days)^5^ | Mean P (1991–2020)^6^ | Mean PET (1991–2020)^7^ |
| --- | --- | --- | --- | --- | --- | --- | --- | --- |
| Canada, CAN | Southwest Manitoba (Manitoba 5) | Statistics Canada | <https://www.statcan.gc.ca/en/start> | 2017–2023 | 261.5 | 202.0 | 470.26 | 462.18 |
| Czech Republic**^1^** | Kraj Vysočina | Czech Statistical Office | https://csu.gov.cz/home | 2017–2023 | 274.2 | 180.3 | 529.08 | 386.30 |
| Slovakia**^1^** | Nitriansky kraj | Statistical Office of the Slovak Republic | [https://slovak.statistics.sk](https://slovak.statistics.sk/) | 2010–2023 | 274.0 | 179.5 | 497.39 | 467.61 |
| Italy, ITA | Catanzaro province, Calabria region | The Italian National Institute of Statistics data warehouse | <https://esploradati.istat.it/databrowser/#/en/dw> | 2017–2023 | 319.0 | 159.0 | 621.27 | 370.36 |
| Portugal, PRT | Norte region | Statistics Portugal | <https://www.ine.pt/xportal/xmain?xpid=INE&xpgid=ine_main> | 2017–2023 | 274.5 | 181.5 | 1022.43 | 540.98 |
| Turkey, TUR | Niğde province, Anadolu Bölgesi region | Turkish Statistical Institute Database | <https://biruni.tuik.gov.tr/medas/?kn=92&locale=en> | 2017–2023 | 304.0 | 212.5 | 472.96 | 716.01 |
| Argentina, ARG**^2^** | General Roca departement, Córdoba province | Agriculture, Livestock and Fisheries Data | <https://datos.magyp.gob.ar/> | 2012–2021 | 301.0 | 153.0 | 598.83 | 957.14 |
| Australia, AUS | The Mallee region, Victoria territory | Grains Research & Development Corporation (GRDC), 2018. Cereal Rye Southern Region - GrowNotes. | https://grdc.com.au/resources-and-publications/grownotes/crop-agronomy/cereal-rye-southern-region-grownotes |  | 232.5 | 209.5 | 310.16 | 1452.63 |
| Country | Rye-production area used for AI | Data source | Website | Years used to define the recent production area | Sowing date (Julian days)^5^ | Harvest date (Julian days)^5^ | Mean P (1991–2020)^6^ | Mean PET (1991–2020)^7^ |
|  |  | Department of Primary Industries and Regions (PIRSA)**^3^** | https://pir.sa.gov.au/ | 2021–2023 |  |  |  |  |
| Germany, DEU**^4^** | Brandenburg federal state | Uhlmann, F., Kleinhanß, W., 2002. Analyse zur Roggenmarktpolitik: alternative Ausgestaltung oder Abschaffung der Roggenintervention? Bundesforschungsanstalt für Landwirtschaft Institut für Marktanalyse und Agrarhandelspolitik, Braunschweig. | https://literatur.thuenen.de/digbib_extern/zi027477.pdf | 1990, 1992, 1995, 2001 | 274.5 | 181.5 | 476.15 | 410.78 |
|  |  | The database of the Federal Statistical Office | <https://www-genesis.destatis.de/datenbank/online/> | 2017–2023 |  |  |  |  |
| Poland, POL | Województwo Wielkopolskie | Statistics Poland | <https://dbw.stat.gov.pl/en/baza-danych> | 2017–2023 | 274.0 | 179.5 | 450.91 | 409.59 |

^1^Aridity index for Czechoslovakia was calculated as a mean aridity index for the Czech Republic (1.383) and Slovakia (1.081).

^2^Available only up to harvest 2021.

^3^Crop and pasture reports.

^4^The agronomical data on rye also include other winter-sown cereals since 2004 in Germany, and hence, also publication with older data only for rye was used.

^5^Sowing and harvest dates were taken from SAGE CropCalendar (<https://sage.nelson.wisc.edu/data-and-models/datasets/crop-calendar-dataset/>), as described by Sacks et al. (2010).

^6^Data from the ERA-5 land database by Muñoz Sabater (2019).

^7^Calculated via the FAO-56 Penman‒Monteith method by Allen et al. (1998).

**Supplementary Table S4**

Post-hoc grouping summary (compact letter display) from Tukey’s *HSD* test (*p* = 0.05, *n* ≥ 4) performed after two-way ANOVA for each trait separately. For each trait, the letters shown for Control and Stressed denote the Tukey grouping(s) assigned to the corresponding variety × treatment mean; means sharing at least one letter are not significantly different. Traits are: grain weight per spike (GW; g), grain number per spike (GN; pcs), thousand-grain weight (TGW; g), harvest index (HI; unitless), straw and leaf weight (SLW; g), aboveground biomass per plant (AB; g), chlorophyll index (CI_F; Dualex units), and grain isotopic composition (δ^15^N; ‰) and ^13^C (δ^13^C; ‰). The significance of the factor variety (V), treatment (T), and their interactive effect (V × T) for each experimental trait and the statistically significant differences among drought stress and the appropriate control treatment for individual rye varieties are shown in Figs. 2–4.

| Variety | GW (g) | | GN (pcs) | | TGW (g) | | HI (unitless) | | SLW (g) | | AB (g) | | CI_F (Dualex units) | | δ^15^N (‰) | | δ^13^C (‰) | |
| --- | --- | --- | --- | --- | --- | --- | --- | --- | --- | --- | --- | --- | --- | --- | --- | --- | --- | --- |
|  | Control | Stressed | Control | Stressed | Control | Stressed | Control | Stressed | Control | Stressed | Control | Stressed | Control | Stressed | Control | Stressed | Control | Stressed |
| Musketeer | abcde | abcd | abcdefg | abcdefg | abcdef | abcdef | abcdefghi | abcdefghi | bcdef | abc | efghijkl | abcd | abcd | abcd | bcdefghi | abcdefg | abcdefghi | cdefghi |
| Prima | abcd | abc | abcdefg | abcdefg | abcdef | abcdef | abcdefghi | abcdefghi | abcde | ab | ghijkl | ab | abcd | abcd | ghi | abcdefg | abcdefg | bcdefghi |
| Stupicke S II | a | abc | ab | abcdefg | abcdef | abcdef | a | abc | ef | def | bcdefghi | bcdefgh | abcd | ab | bcdefghi | abc | efghi | hi |
| Cinquecento | abcd | abc | abcdef | abcdefg | abcdef | abcdef | abcd | abcde | f | abcde | efghijkl | abcdef | bcd | abcd | ghi | abcdefghi | bcdefghi | cdefghi |
| Montalegre | abc | a | abcdefg | abcdefg | abcdef | abcd | abcdefg | abcde | abcd | abcd | cdefghijk | abcdefg | abcd | a | fghi | abcdefg | abcdefghi | i |
| Caudar | a | ab | a | abc | bcdef | abcdef | ab | abcde | cdef | abcd | efghijkl | abcdef | abcd | abcd | defghi | abcdefghi | bcdefghi | ghi |
| Choigue | a | ab | abcdef | abcdefg | abcdef | abcde | abcde | abcdefg | abcd | abc | defghijkl | abcdefg | abcd | abc | hi | abcdefghi | abcdefg | bcdefghi |
| Naico | bcde | abcd | defg | abcdefg | abcdef | abcdef | cdefghi | abcdefghi | bcdef | abcd | hijkl | abcdefg | abcd | abcd | defghi | abcdefghi | abcde | fghi |
| Elbon Gator 17 | abcde | ab | defg | bcdefg | abcde | a | bcdefghi | abcde | abcd | abcd | defghijkl | abcdef | abcd | ab | abcdefgh | a | abcd | bcdefghi |
| Wrens-5 | abcde | ab | fg | bcdefg | abc | ab | abcdefghi | bcdefghi | abcde | a | abcdefgh | a | abcd | abcd | bcdefghi | abcd | abcdefg | bcdefghi |
| KWS Binntto | e | abcde | g | cdefg | f | abcdef | hi | defghi | bcdef | abcd | kl | bcdefghij | cd | abcd | abcdefghi | ab | abcdef | abcdefg |
| Variety | GW (g) | | GN (pcs) | | TGW (g) | | HI (unitless) | | SLW (g) | | AB (g) | | CI_F (Dualex units) | | δ^15^N (‰) | | δ^13^C (‰) | |
|  | Control | Stressed | Control | Stressed | Control | Stressed | Control | Stressed | Control | Stressed | Control | Stressed | Control | Stressed | Control | Stressed | Control | Stressed |
| KWS Vinetto | cde | abcde | efg | bcdefg | def | abcdef | fghi | cdefghi | abcd | ab | ijkl | abcdefg | d | abcd | efghi | abcde | abc | abcdefg |
| SU Cossani | cde | abcde | fg | defg | bcdef | abcdef | ghi | efghi | abcd | abc | jkl | bcdefghij | bcd | abcd | bcdefghi | abcd | ab | abcdefg |
| SU Performer | de | abcde | g | abcdefg | abcdef | abcdef | i | cdefghi | abcd | abcd | l | cdefghij | bcd | abcd | abcdefghi | abcdefg | a | abcdefg |
| Conduct | abcde | ab | abcdefg | ab | ef | abcdef | abcdefghi | abcde | bcdef | abcd | ghijkl | abcde | bcd | abcd | hi | abcdefghi | abcdefg | defghi |
| Dukato | ab | abcd | abcd | abcde | bcdef | cdef | abcde | abcdef | bcdef | abcde | abcdefg | abcdefg | abcd | abcd | i | cdefghi | bcdefghi | efghi |
| Inspector | abcde | abc | abcdefg | abcdefg | ef | abcdef | bcdefghi | abcdefgh | abcd | abcd | ghijkl | abcdefg | abcd | abc | fghi | abcdefgh | abcdefg | bcdefghi |
| Matador | abcde | abcd | fg | abcdefg | abcdef | abcdef | bcdefghi | abcdefghi | bcdef | abcd | efghijkl | abc | abcd | abc | bcdefghi | abcdefg | abcdefg | abcdefghi |
| Dankowskie Amber | abcde | abcd | abcdefg | abcdefg | f | abcdef | bcdefghi | abcdef | bcdef | bcdef | fghijkl | abcdefg | abcd | abc | hi | abcdef | abc | bcdefghi |
| Dankowskie Rubin | abcde | abcd | abcdefg | abcdefg | ef | abcdef | abcdefghi | abcdefghi | bcdef | abcd | fghijkl | abcdefg | abcd | abcd | hi | abcdef | abcdefgh | bcdefghi |


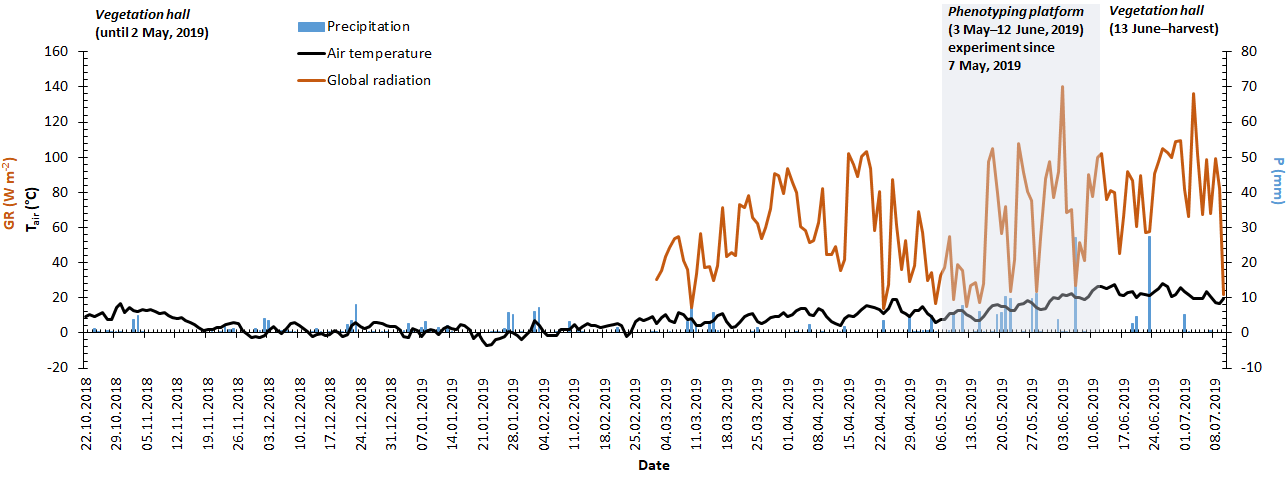


**Supplementary Fig. S1** The course of mean daily air temperatures (°C; measured at 2 m above ground level at 10-minute intervals using a Minikin T3 sensor, EMS Brno, Czech Republic) and mean daily global radiation (W m^-2^; measured at the stand level at 1-minute interval using a Minikin RT Global Radiation Sensor, EMS Brno, Czech Republic) during the placement of experimental plants in the vegetation hall in Brno are presented. Precipitation (mm; Tripping Bucket Rain Gauge model 52202, R.M. Young Company, Traverse City, Michigan, USA) was measured in the nearby arboretum of Mendel University in Brno (approximately 450 m from the vegetation hall). The total precipitation recorded in the open area of the arboretum during plant cultivation was 170 mm. The total additional irrigation of plants per vegetation was 87 mm.


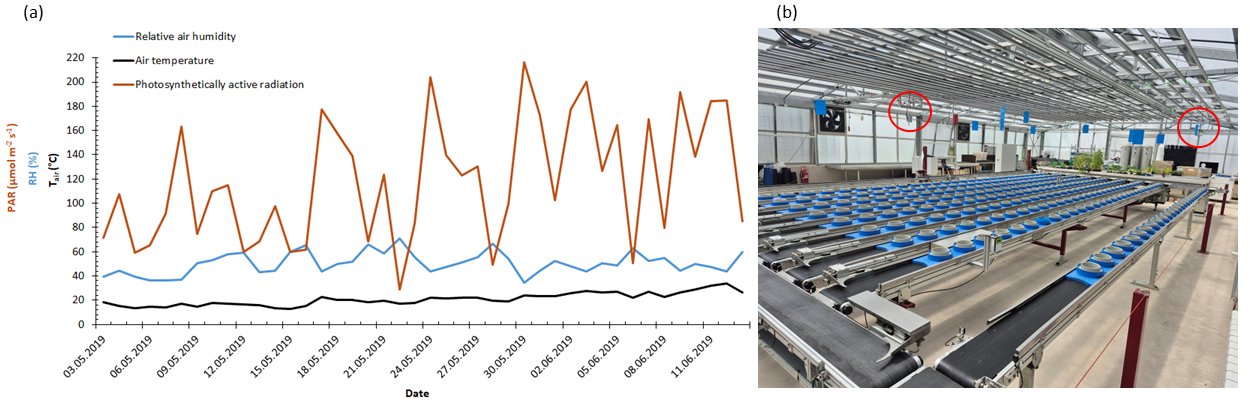


**Supplementary Fig. S2** **a** The course of mean daily air temperature (°C), mean daily relative air humidity (%), and mean daily photosynthetically active radiation (µmol m^-2^ s^-1^) measured in the greenhouse of Photon Systems Instruments, Ltd., in Drásov at 1-minute intervals above the plant canopy using two Multisensor V130 probes (Photon Systems Instruments, Ltd., Drásov, Czech Republic). The placements of the probes in the greenhouse of Drásov with the phenotyping platform used in this study are shown in the right panel **b**. The mean values of the measured air temperatures, relative air humidity, and photosynthetically active radiation are calculated by central computer software to control the phenotyping platform and are stored directly as mean values instead of values of individual probes.
